# Combined evaluation of CAC score and myocardial perfusion imaging in patients at risk of cardiovascular disease: where are we and what do the data say

**DOI:** 10.1007/s12350-023-03288-2

**Published:** 2023-05-10

**Authors:** Teresa Mannarino, Adriana D’Antonio, Roberta Assante, Emilia Zampella, Valeria Gaudieri, Mario Petretta, Alberto Cuocolo, Wanda Acampa

**Affiliations:** 1 Department of Advanced Biomedical Sciences, University “Federico II” of Naples, Via Pansini 5, 80131 Naples, Italy; 2 IRCCS Synlab SDN, Via Gianturco 113, 80142 Naples, Italy

**Keywords:** Hybrid imaging, MPI, atherosclerosis, CAD

## Abstract

Advances in the prevention and treatment of cardiovascular disease (CVD) over the last decades have led to a marked reduction in mortality for CVD. Nevertheless, atherosclerosis leading to coronary artery disease and stroke remains one of the most common causes of death in the world. The usefulness of imaging tests in the early identification of disease led to identify subjects at major risk of poor outcomes, suggesting risk factor modification. The aim of this article is to analyze the state of art of combined imaging in patients at risk of CVD referred to MPI evaluation, to highlight the present and potential features able to provide incremental prognostic information to help clinicians in patient management and to reduce adverse events.

**Supplementary Information:**

The online version contains supplementary material available at 10.1007/s12350-023-03288-2.

Cardiovascular disease (CVD) represents one of the main causes of morbidity and mortality in the world. Atherosclerosis leading to coronary artery disease (CAD) and stroke is one of the most common causes of death in the United States and in Europe. CVD is responsible for 4.1 million deaths in Europe accounting to 47% and 39% of all deaths in females and males, respectively.^1^ The advances in both prevention and treatment of CVD over the last decades have led to a marked reduction in age-adjusted mortality. Nevertheless, CVD remains one of the most important expenditure items in public health, being responsible of several hospital admissions. An analysis by the American Heart Association (AHA) noted that the direct costs of cardiovascular health care were projected to triple by 2030 to an estimated cost of 818 billion.^2^ Therefore, it has become imperative to develop an effective strategy for preventing CVD. The earlier identification of CVD will permit the identification of the disease in its subclinical phase or in early symptomatic stage, providing opportunity to institute risk factor modification and to adjust medical therapy before the development of more advanced disease, clinical complications, and/or occurrence of adverse cardiac events.

## Global risk factor algorithms and the emerging role of coronary artery calcium score

Considering the presence of a high prevalence of CVD risk factors among patients who develop clinical disease, interest has focused on developing global risk factor algorithms for predicting CVD. Several algorithms were proposed during the late 1990s and 2000s. The Systematic Coronary Risk Evaluation project^3^ formulated a CVD risk estimation algorithm (HEARTSCORE) that has been adopted by the Joint European Societies’ guidelines on CVD prevention. Some investigators developed other specific algorithms as QRISK^4^ and ASSIGN.^5^ The first global validated algorithm for predicting CVD was Framingham score (FRS)^6^ and it remains one of the most reliable tools in the evaluation of cardiovascular risk factors. The FRS was born as a single multivariable risk assessment tool that would enable physicians to identify high-risk candidates for all initial atherosclerotic CVD events. The utilization and clinical value of such approach are limited by the fact that neither the chronicity of the disease nor its magnitude are considered. However, the evaluation of this score and related risk stratification of the patients led to an initial strategy of patient management: subjects with a high 10-year risk would worth aggressive risk factor modification while intermediate risk would require further testing to evaluate their risk status based on other measures of cardiac risk. Of note, the “intermediate risk” category is quite composite: it contains both patients for whom more aggressive therapy might be indicated, and it also contains lower risk individuals who might be managed with less aggressive therapy and/or lifestyle measures. This recognition has motivated research to identify testing that could offer greater discrimination of higher and lower risk patients within the intermediate risk group. In this scenario, the role of coronary artery calcium (CAC) score has become increasingly relevant,^7^ offering the opportunity to directly evaluate the presence of coronary calcium in the arteries leading to a further risk stratification for CVD.^8^ Agatston et al.^9^ proposed a quantitative method to evaluate coronary calcific based on Hounsfield units. Many published data used this method for the quantitative evaluation of calcium content in the coronary arteries by NCCT.^10–20^ Recent published guidelines have clearly outlined the technical aspects of CAC measurement and its clinical value.^21^

The measurements of CAC scores have been demonstrated of great clinical utility in risk stratify patients at intermediate risk. Shaw et al.^22^ showed that CAC score is a potent predictor of future cardiac events. In particular, the mild increase in the CAC score, even in the range of only 1 to 10, demonstrated to be enough to at least double the risk of adverse clinical events as compared to subjects with zero CAC score. Accordingly, it has also been demonstrated that patients at intermediate risk of CAD with calcium score of 1 to 100 remained below 2% estimated risk of cardiovascular events for 18 months, while it has been suggested that patients with zero CAC score may have a warranty period not lower than 4 to 5 years.^23^ Interestingly, other studies demonstrated the significant incremental value of CAC score over other traditional screening methods. Important analysis from the MESA (Multi-Ethnic Study of Atherosclerosis) in 1,330 subjects, followed up for a mean of 7.5 years, showed that CAC score offered a high degree of net reclassification improvement (NRI) for the prediction of incident CVD compared to a low NRI by the other screening modalities.^24^ Therefore, it appears justified to use CAC screening to identify intermediate risk, by traditional risk factors, for which the treatment of atherosclerotic disease should be indicated. CAC has shown a 100% negative predictive value (NPV), for ruling out significant coronary narrowing,^25^ but different studies have disputed this result because a functionally significant stenosis is possible even in the absence of CAC.^26^ To provide an accurate evaluation of CAC scoring, an ECG gated noncontrast CT (NCCT) evaluation is required to avoid motion artifact, using specific reconstruction methodologies by slice thickness.

The guidelines for coronary artery calcium scoring chest CT scans, within CAC reporting section, recommended in Class I that CAC should be evaluated and reported on all noncontrast chest CT examination using a scoring methodology.^27^ The coronary artery calcium scoring methodologies included ECG gated Agatston scoring, nongated Agatston scoring, nongated ordinal scoring, and visual assessment. It has been demonstrated in fact that visual evaluation of CAC in attenuation correction nongated CT scan has high agreement with Agatston score.^28^ Automated quantification of CAC is feasible in nongated noncontrast-enhanced CT with good reliability and agreement when compared to reference scores.^29^ More recently, it has been demonstrated that CAC scoring of CTAC can be performed routinely without modification of PET protocol and added radiation dose.^30^ Nongated Agatston scoring, obviously, requires a computer analysis with specific software.^30,31^ The recent introduction of specific fast software allows to semi-automatically calculate Agatston score by a CT acquisition, placing a region of interest around the calcific lesion and consequently summing the score in the three major coronary vessels (i.e., Vitrea FX, VScore analysis software, Vital Images, Minnetonka, USA; Cardiac Suite, Cedars Sinai Medical Center, CA, USA). A voxel is identified as belonging to a calcified lesion if the voxel attenuation is above a known threshold (usually 130 Hounsfield Units) and at least 3 voxels are contiguous, to ignore the structures < 1 mm^2^, minimizing the effect of the image noise (spatial threshold). In the latest years, sophisticated software with deep learning-based algorithms to calculate CAC has been already developed,^30–34^ with the aim to improve the automatization of the calculation of atherosclerotic burden. Although the real impact of this tool in routine clinical practice needs still to be addressed, the recent results are strongly encouraging. In particular, it has been demonstrated that a deep learning algorithm for CAC scoring showed good agreement with manual scoring even in the evaluation of nongated attenuation correction noncardiac dedicated CT.^34^

## Key points from published evidence by CAC data

1. Global risk factor algorithms are clinically used for predicting CVD to an initial strategy of patient management.
2. ECG gated NCCT using a quantitative evaluation of coronary calcium by Agatston score was widely used to perform an accurate evaluation of the amount of calcium to predict CVD risk.
3. The guidelines for coronary artery calcium scoring chest CT scans recommended (Class I) that CAC should be evaluated and reported on all noncontrast chest CT examination using one of the scoring methodology available: ECG gated Agatston scoring, nongated Agatston scoring, nongated ordinal scoring, and visual assessment.

## Combined evaluation of CAC score and MPI

### Two different imaging techniques and modalities

The combined evaluation of CAC score and myocardial perfusion has been widely used in the evaluation of patients with suspected CAD and in risk stratifying such patients, referring to a specific therapeutic approach. A huge spectrum of published articles is available combining data by a separate evaluation of CAC score and perfusion, using two different imaging techniques and modalities. Table 1 lists some studies providing diagnostic and prognostic data using a dedicated CT scan (gated or ungated) with an Agatston quantitative classification for CAC, with different points score system, and a SPECT acquisition for perfusion assessment.

**Table 1:** Combined evaluation of CAC score and MPI: diagnostic and prognostic data using a dedicated CT scan

Study	Gated/nongated CT	Agatston or visual score	Clinical endpoints	N° patients	MPI method
Separate evaluation with dedicated CT					
He et al.^10^ 2000	Gated	0, 1–10, 11–100, 101–399, ≥ 400	Diagnostic: CAC and ischemia in suspected CAD	411	SPECT
Moser et al.^11^ 2003	Gated	0–100, 101–400, > 400	Diagnostic: CAC, risk factors and ischemia in suspected CAD	102	SPECT
Anand et al. ^12^ 2004	Gated	101–399, ≥ 400	Diagnostic: CAC and ischemia in suspected CAD	220	SPECT
Berman et al.^38^ 2004	Gated	0, 1–9, 10–99, 100–399, 400–999, ≥ 1000	Diagnostic: CAC and ischemia in suspected CAD	1195	SPECT
Nishida et al.^37^ 2005	Nongated	0, > 0	Diagnostic: CAC and ischemia in symptomatic suspected CAD	83	SPECT
Anand et al.^14^ 2006	Gated	101–400 401–1000, ≥ 1000	Diagnostic: CAC and ischemia in diabetic patients	180	SPECT
Blumenthal et al. ^15^ 2006	Gated	0, 1–10, 11–100, 101–399, ≥ 400	Diagnostic: CAC, ischemia and risk factors in subjects with family history of CAD	260	SPECT
Rosman et al.^16^ 2006	Gated	0, 1–99, 100–399, 400–999, ≥ 1000	Diagnostic: CAC and abnormal MPI in suspected CAD	126	SPECT
Ramakrishna et al.^17^ 2007	Gated	0, 1–10, 11–100, 101–400, > 400	Prognostic: CAC and SSS in suspected CAD	835	SPECT
Ho et al.^18^ 2007	Not specified	0–10, 11–100, 101–400, 401–1,000, > 1,000	Diagnostic: CAC and ischemia in suspected CAD	703	SPECT
Rozanski et al.^19^ 2007	Not specified	0, 1–9, 10–99, 100–399, 400–999, ≥ 1000	Prognostic: CAC and ischemia in suspected CAD	1153	SPECT
von Ziegler et al.^36^ 2012	Gated	0, < 10, < 100, < 400	Diagnostic: CAC and ischemia in hemodynamically relevant CAD	351	SPECT
Chang et al.^39^ 2015	Not specified	≤ 10, 11–100, 101–400, > 400	Prognostic: CAC, exercise treadmill test and ischemia in suspected CAD	946	SPECT
Nappi et al.^35^ 2018	Gated	0, 1–300, > 300	Prognostic: CAC, coronary CT and ischemia in suspected CAD	156	SPECT
Trpkov et al.^40^ 2021	Gated	Absent, equivocal, present, extensive	Prognostic: CAC and MPI in suspected or known CAD	4720	SPECT

*CAC*, coronary artery calcium; *CAD*, coronary artery disease; *MPI*, myocardial perfusion imaging; *SSS*, summed stress score

From these data, it emerged that the major predictors of events in patients with suspected CAD are the extent of atherosclerotic burden, assessed by CAC, and the extent and severity of stress-induced myocardial ischemia assessed by MPI.^35–37^ In particular, the evaluation of CAC content in association with myocardial perfusion showed an improvement in the specificity and positive predictive value to detect CAD, especially in patients with a CAC score value more than zero.^36^ It should be also considered that subclinical atherosclerosis may be also frequently present in patients with normal myocardial perfusion, as well as a normal MPI does not necessarily exclude significant coronary stenosis. Indeed, different studies demonstrated that subclinical atherosclerosis is quite common among patients who are referred to cardiac stress testing showing abnormal CAC score in the absence of myocardial perfusion abnormalities. Berman et al.^38^ demonstrated that among 1119 patients with normal MPI studies, 78% had evidence of CAC and in 30% of patients CAC score was > 400, indicating extensive grade of atherosclerosis. In a prospective observational study conducted by Chang et al.,^39^ 988 asymptomatic or symptomatic patients were evaluated, to define the relative value of CAC score, exercise treadmill testing (ETT), and SPECT variables in predicting long-term risk stratification. From these data it emerged that CAC score, by electron beam CT and evaluated using Agatston criteria, significantly improved long-term risk stratification beyond FRS, ETT, and SPECT results across the spectrum of clinical risk and, importantly, even among those who are currently considered appropriate candidates for functional testing or have low-risk functional test results. Similarly, visual CAC resulted in an independent predictor of cardiac events over the results of SPECT MPI.^40^

### One imaging hybrid technique and different modalities

As well known, the introduction of hybrid imaging single-photon emission computed tomography (SPECT)/CT and positron emission tomography (PET)/CT with different radiotracers allows to perform a combined evaluation of perfusion and both functional and structural abnormalities. This approach has been used also in nuclear cardiology, with the different radiotracers available, for the detection of obstructive CAD and for risk stratification in a huge spectrum of patients with suspected or known CAD, as those at intermediate-high CAD risk.^41–44^ Table 2A lists some studies providing diagnostic and prognostic data of combined CAC and perfusion evaluation in patients with suspected CAD using a single hybrid imaging but different methodologic approaches. In particular, the combined evaluation of CAC and perfusion was performed using an additional CT to the SPECT/CT or PET/CT acquisition exposing patients to an additional radiation dose. These studies confirmed that many patients with normal perfusion showed the presence of CAC, and, on the other hand, the absence of CAC does not completely exclude flow-limiting CAD^45^ in patients performing a third CT scan with retrospective ECG gating for CAC scoring after myocardial perfusion imaging by PET/CT. In particular, by SPECT/CT Engbers et al.^46^ demonstrated that CAC score and SPECT findings are independent predictors of events in symptomatic patients at low-intermediate risk. However, a large spectrum of data have been published to outline the prognostic power of a combined evaluation by PET/CT imaging.^42,47–50^ The important value of a combined evaluation by PET/CT imaging is provided by the possibility to perform a dynamic acquisition imaging. In particular, a combined evaluation of myocardial perfusion and atherosclerotic burden by CAC score was tested by dynamic 82-Rubidium (^82^Rb)-PET/CT, using all the perfusion parameters available by this imaging technique, including myocardial perfusion reserve (MPR) evaluation. Different studies tried to address the potential incremental prognostic utility of MPR over standard MPI using PET/CT, with various results. It was clearly demonstrated that, although in symptomatic patients with normal MPI, MPR, indicating coronary microvascular dysfunction, decreased with increasing levels of CAC, only MPR but not CAC provides significant incremental risk stratification over clinical risk score for the prediction of major adverse cardiac events.^47^ These results were obtained performing a separate CT scan for CAC scoring during breath-hold.^47^ Thus, in patients with suspected CAD, the combined evaluation of CAC and MPI, including functional parameters like MPR, may incrementally add to their correct classification over risk categories, improving the diagnostic power of the imaging techniques.

**Table 2:** Combined evaluation of CAC score and MPI: diagnostic and prognostic data using hybrid imaging

Study	Gated/nongated CT	Agatston or visual score	Clinical endpoints	N° patients	MPI method
(A) Additional dedicated CT					
Schepis et al.^20^ 2007	Gated	< 10, 11–100, 101–400, 401–1000, > 1000	Diagnostic: CAC and abnormal MPI in intermediate risk of CAD	77	SPECT/CT
Schenker et al.^45^ 2008	Gated	0, 1–399, 400–999, ≥ 1000	Prognostic: CAC and ischemia in intermediate risk of CAD	621	PET/CT
Bybee et al.^53^ 2010	Gated	0, > 0	Diagnostic: CAC and normal MPI in suspected CAD	760	PET/CT
Naya et al.^47^ 2013	Gated	0, 1–399, ≥ 400	Prognostic: CAC and CFR in suspected CAD	901	PET/CT
Brodov et al.^41^ 2015	Not specified	0, 1–99, 100–399, ≥ 400	Diagnostic: regional CAC and ITPD in suspected CAD	152	PET/CT
Assante et al.^48^ 2016	Nongated	0 < 1–99, 100–399, ≥ 400	Diagnostic: CAC and CFR in suspected CAD	637	PET/CT
Engbers et al.^46^ 2016	Gated	0, 1–99, 100–399, 400–999, ≥ 1000	Prognostic: CAC and abnormal MPI in low-intermediate risk of CAD	4897	SPECT/CT
Assante et al.^59^ 2017	Nongated	0, 1–399, ≥ 400	Prognostic: CAC and CFR in low-intermediate risk of CAD	436	PET/CT
Assante et al.^52^ 2017	Nongated	Quartiles	Diagnostic: CAC and CFR in diabetic and nondiabetic patients	766	PET/CT
Sharma et al.^13^ 2019	Gated	≤ 216, > 216	Prognostic: CAC and TPD in symptomatic suspected CAD	655	SPECT/CT
Aljizeeri et al.^50^ 2021	Gated	0, 1–99, 100–399, ≥ 400	Prognostic: CAC and MPR in suspected CAD	4067	PET/CT
Miller et al.^49^ 2022	Gated	0, 1–99, 100–399, 400–999, ≥ 1000	Prognostic: CAC and ITPD in suspected CAD	2507	PET/CT
Patel et al.^51^ 2022	Gated	0, 1–99, 100–399, ≥ 400	Prognostic: CAC, abnormal MPI and MPR in suspected CAD	5983	PET/CT
(B) Attenuation correction CT					
Esteves et al.^54^ 2008	Nongated	Visual: Present vs Absent	Diagnostic: CAC and normal MPI in chest pain	80	PET/CT
Fathala et al.^55^ 2011	Nongated	Visual: Present vs Absent	Diagnostic: CAC and ischemia in cancer patients with suspected CAD	157	PET/CT
Zampella et al.^56^ 2018	Nongated	< 100, ≥ 100	Diagnostic: regional CAC, ITPD and CFR in suspected CAD	113	PET/CT
Zampella et al.^57^ 2019	Nongated	< 300, ≥ 300	Prognostic: regional CAC, ITPD and CFR in suspected CAD	206	PET/CT
Dekker et al.^31^ 2020	Nongated	0, 1–100, 101–300, ≥ 301	Diagnostic: CAC score and ischemia in suspected CAD	150	PET/CT

*CAC*, coronary artery calcium; *MPI*, myocardial perfusion imaging; *CAD*, coronary artery disease; *CFR*, coronary flow reserve; *MPR*, myocardial perfusion reserve; *ITPD*, ischemic total perfusion deficit; *TPD*, total perfusion defect

**Figure 1:**
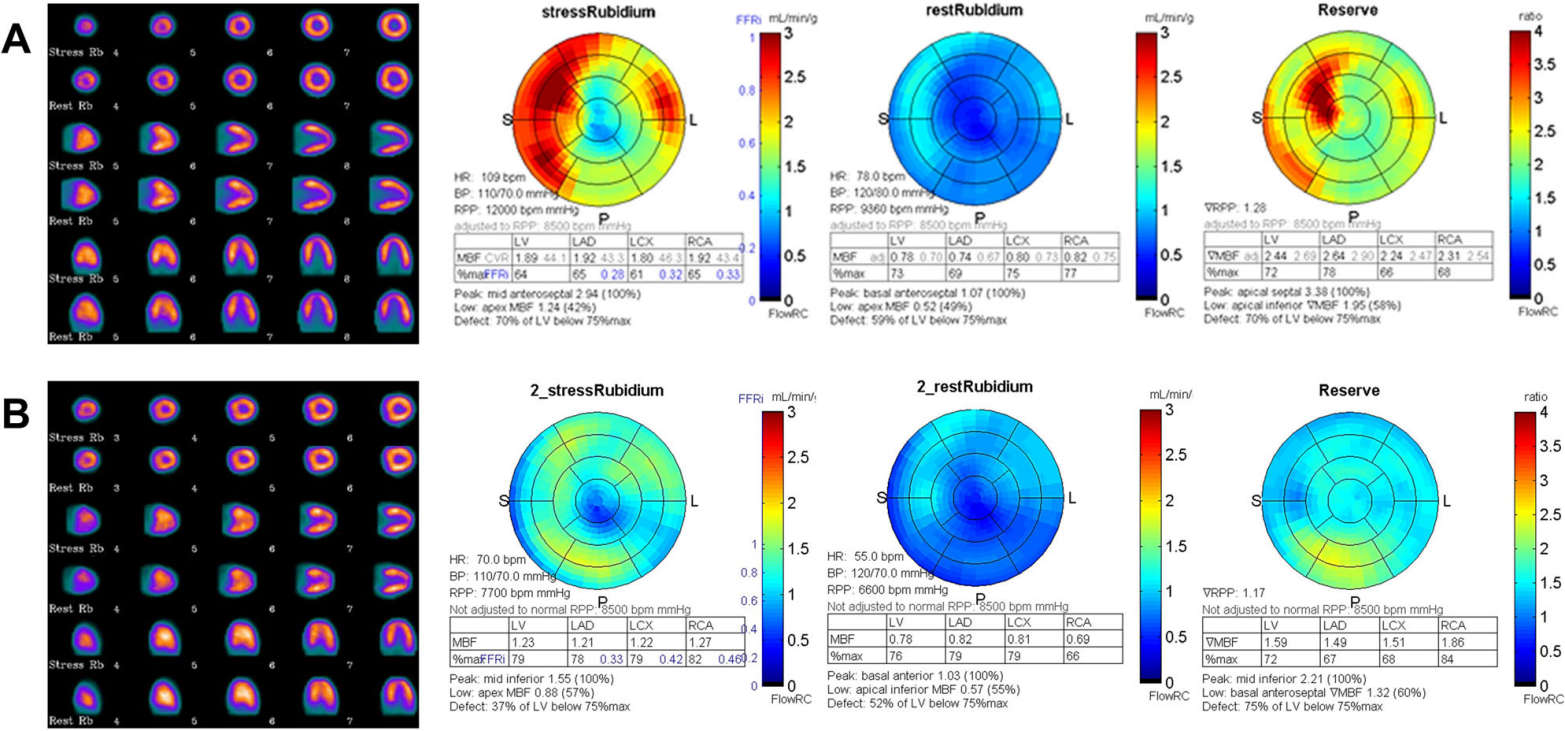
^82^Rubidium PET/CT perfusion imaging and myocardial perfusion reserve in nondiabetic (**A**) and diabetic (**B**) patients with CAC score 0

**Figure 2:**
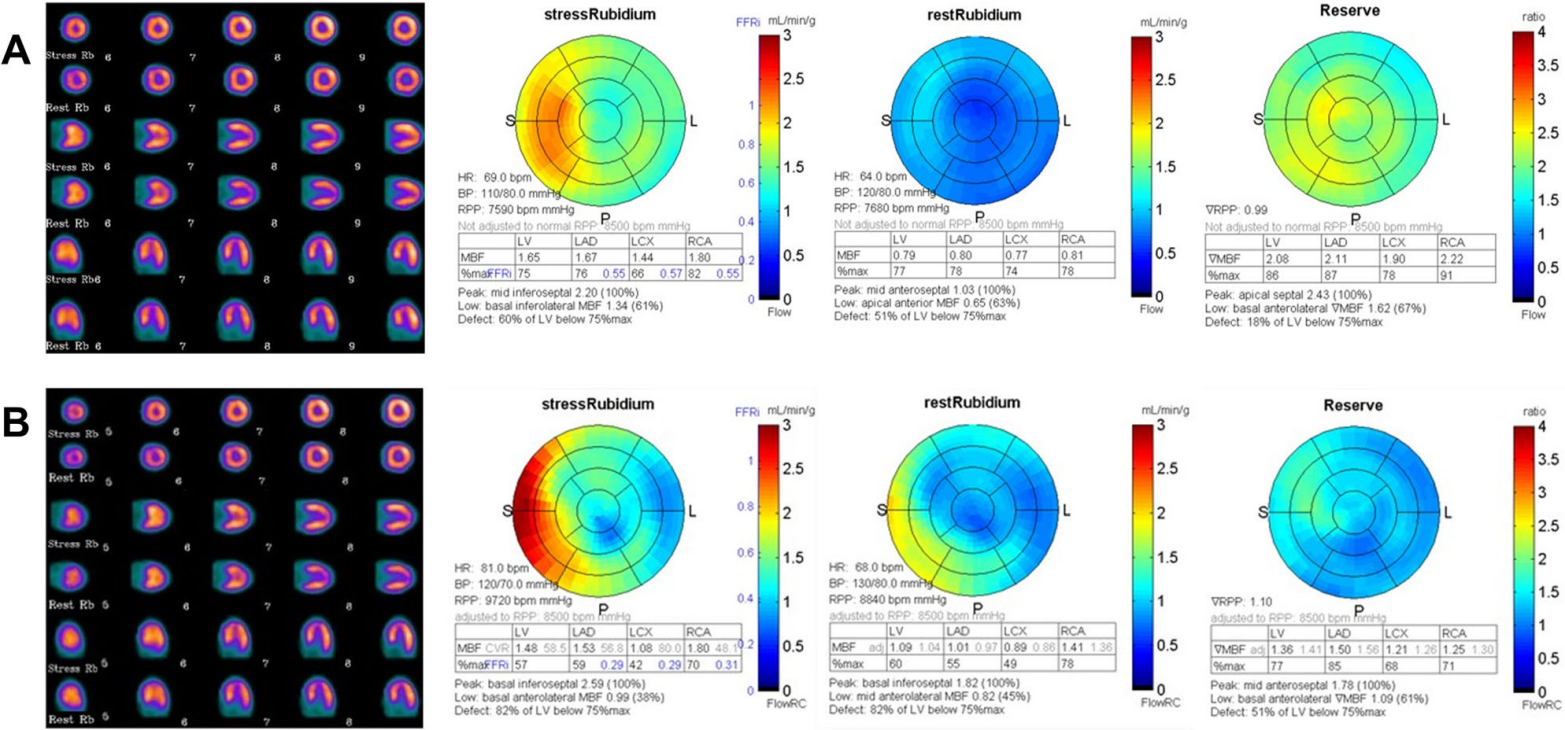
^82^Rubidium PET/CT perfusion imaging and myocardial perfusion reserve in nondiabetic (**A**) and diabetic (**B**) patients with CAC score > 400

Assante et al.,^48^ in a large cohort of patients with suspected CAD, used quantitative ^82^Rb PET/CT imaging using an additional CT, and assessed the relationship between CAC score and coronary vascular function evaluating if CAC score can predict a myocardial dysfunction independently from conventional coronary risk factors. The results showed that MPR progressively decreased with increasing CAC score levels, accordingly with Naya’s results.^47^ Noteworthy, only the group of patients with a CAC score > 400 had an average MPR below normal value. Thus, coronary atherosclerotic burden and vascular function seem to be two different entities with distinct etiology and alternative potential therapeutic strategies. It should be considered that recent published literature is quite controversial on the benefit of CAC evaluation over MPR. In particular, Miller et al.^49^ demonstrated that both CAC and ischemic TPD are independently associated with MACE even after incorporating information regarding MPR. Some authors otherwise highlighted the incremental prognostic value of CAC and MPR over clinical and MPI variables.^50^ Conversely, some other data demonstrated the independent association between CAC and death, but without an incremental prognostic value of CAC over PET imaging results including MPR.^51^ Also, in selected categories of patients, such as diabetic patients, a significant decrease in MPR with increasing CAC score quartile was demonstrated, which was also observed in nondiabetic patients. However, the presence of diabetes has been demonstrated to be associated with significantly lower MPR across CAC quartile categories.^52^ Figures 1 and 2 show coronary vascular function in nondiabetic and diabetic patients according to the presence or absence of CAC. The two cases show that although there was absence of perfusion abnormalities, the presence of diabetes and a high value of CAC score are related to impaired MPR.

Despite these important results, a real challenge could be to understand how some of the prognostic information provided by nuclear imaging methods can be translated into an effective clinical decision and significantly changing clinical management. Appropriately, Bybee et al.^53^ evaluated CAC score, using a calcium scoring CT, through different FRS categories in patients with no history of CAD and negative positron emission tomography (PET)/CT, showing that among patients with low FRS, 57% had CAC. As well, in the intermediate FRS group, 64% had CAC (26% with a score > 100) and in the high-risk group 82% had CAC (51% with a score > 100). Adding this anatomic imaging result to normal physiologic findings may likely help the referring physician to guide the subsequent medical management for the prevention of ischemic heart disease events. In fact, increasing CAC score was associated with a greater likelihood of initiation or optimization of medical therapy for CAD.^53^ In patients not receiving statin therapy prior to the study, those with CAC were more likely to be initiated on statin therapy compared to those without CAC. The increasing Agatston score was independently associated with initiation or optimization of medical therapy for CAD in the multivariate model. This study revealed that, according to the presence of coronary calcification, there was a significant under treatment for CAD prior to PET/CT MPI in patients with subclinical CAD. For example, only 41.5% of patients found to have a CAC score 1 to 399, and 47.7% of patient found to have a CACS > 400 were receiving statin therapy prior to the PET/CT MPI. Similarly, only 43.7% of patients were found to have a CAC score 1 to 399 and 51.4% of patients with a CACS > 400 were receiving aspirin therapy prior to the study. Subsequent changes or recommendations for optimization of medical therapy for CAD were more likely to occur in those found to have CAC compared to those without CAC.^53^ At this time more data need to be provided to assess the clinical real benefit combining the different parameters available by the imaging modalities available.

### One imaging hybrid technique for all the different parameters

All these data available, despite were provided by a hybrid camera, used an additional CT for the evaluation of CAC score parameters. As discussed, recent data clearly outlined that ubiquitous CTAC, available in patients referred to MPI by/CT PET studies, can be used for quantitative CAC analysis in clinical evaluation.^30^ These findings should have a great impact in the assessment of individual cardiovascular risk of patients referred to MPI by PET/CT, potentially affecting patient decision-making.^30^ Moreover, as shown in Table 2B, till date only few clinical studies evaluated a combination of atherosclerotic burden and myocardial perfusion abnormalities using low-dose attenuation correction CT scan by PET/CT MPI.^54–58^ In particular, an extremely high negative predictive value was observed, both in symptomatic patients referred to the chest pain unit and, as well, in the preoperative assessment of cancer patients at intermediate risk of CAD,^54,55^ as already demonstrated in other published data.^59^ The reliability of not detectable CAC score in predicting normal myocardial perfusion may be of great clinical impact in the setting of hybrid modality examination with a low-dose CT, instead of performing an additional dedicated gated CT, reducing patient radiation exposure, not requesting specific patient preparation, ruling out possible ischemia and unnecessary further testing. On the other hand, these studies confirm that in the wide spectrum of the atherosclerotic disease, the presence of coronary calcification does not correspond to a unique perfusion pattern and consequently to a unique risk category. This is one of the reasons why hybrid modality imaging is so attractive also in a diagnostic phase. The full potential of combining calcium and perfusion evaluation by single examination has been investigated also in a regional analysis. Specifically, Zampella et al.^56^ have clearly outlined that adding quantitative evaluation of regional hyperemic MBF and MPR to CAC score and ischemia in per-vessel analysis is able to provide incremental diagnostic value in identifying the presence of obstructive CAD. Similarly, Dekker et al.^31^ found that the diagnostic accuracy of MPI to discover obstructive CAD increased 4% when adding the values of the automated calculation of CAC score. Thus, the diagnostic and clinical benefits of combined CAC score and myocardial perfusion and functional parameters, in a single imaging procedure, have been assessed.^54–58^ These results could be extremely interesting considering that calcium deposits do not completely reflect overall disease activity within coronary circulation and direct measures of coronary vasodilator function may be more powerful measures of CAD risk. Accordingly, the extent of coronary calcification and presence of coronary vascular dysfunction are both associated with an increased risk of adverse cardiac events and are also able to predict lesion-related outcome in patients with suspected CAD.^57,59^ Thus, it is reasonable to hypothesize that a combination of coronary calcium and vascular function assessment, also in the single vessel, may provide information that could possibly help to modify therapeutic strategies in specific subset of patients. Moreover, combining the assessment of overall these parameters in a unique technique could be effective also in terms of cost-effectiveness. However, additional features obtained by combined evaluation by SPECT/CT or PET/CT MPI can be observed for diagnostic and prognostic purposes. It should be considered that, using hybrid imaging procedures like stress gated PET/CT and SPECT/CT, different functional parameters can be assessed and evaluated, also in specific subsets of patients helping in the patient-oriented risk assessment.

## Key points from published evidence by combined CAC and perfusion evaluation

1. The combined evaluation of CAC score and myocardial perfusion has been widely used in risk stratify patients with suspected CAD to refer to a specific therapeutic approach.
2. Most published data available performed a separate evaluation of CAC score and perfusion abnormalities, using two different imaging techniques and modalities.
3. As major predictors of events in patients with suspected CAD resulted in the extent of atherosclerotic burden, assessed by CAC, and the extent and severity of stress-induced myocardial ischemia assessed by MPI.
4. Ubiquitous CTAC, available in patients referred to MPI by PET/CT studies, can be used for quantitative CAC analysis.
5. Despite the diagnostic and clinical benefits of combined CAC score and myocardial perfusion and functional parameters, in a single imaging procedure, have been assessed, this approach needs to be implemented.
6. All MPI modalities performed using a hybrid camera SPECT/CT or PET/CT should provide CAC evaluation combined to the other data available by this procedure as perfusion and blood flow parameters.
7. More data, also in terms of clinical trials, need to be provided to define how a combination of the different parameters by nuclear procedures could help in the risk stratification process.

## Conclusion and future directions

The choice of the most useful imaging techniques in some categories of patients is still challenging, as in patients at intermediate risk of CAD. To the state of art, combined imaging has already demonstrated to be extremely useful in patients risk stratification and various tools are available to help clinicians in preventing and predicting outcomes of CVD in selected categories of patients. Nevertheless, more data for specific endpoints need to be conducted in the setting of patients at risk of CVD and of CV events. Moreover, an additional important endpoint to be reached is also the improvement of cost-effectiveness of imaging-guided heal. The ideal test should be able to modify patient therapeutic strategy without affecting management costs, thus further studies are necessary to measure the correct impact of imaging on CVD prevention and treatment. Thus, future direction should help us to understand and determining how to combine all the variables available to obtain a more patient-oriented risk stratification. In this setting, artificial intelligence could help us to obtain a real combination of clinical and procedural clinical information. As previously discussed, in the latest years, sophisticated software with deep learning-based algorithms to calculate CAC has been already developed.^30–34^ However, further effort would be hopeful to develop algorithms to combine all the variables obtained by an hybrid imaging. A long way walking and specific trials should be designed to demonstrate the clear benefit also in term of cost-effectiveness to refer which patients to which procedure leading to a real patient-centered imaging according to risk stratification.

### Supplementary Information

Below is the link to the electronic supplementary material.

Supplementary file1 (PPTX 209 KB)

Supplementary file2 (MP3 7151 KB)

## Abbreviations

CVD: Cardiovascular disease
CAD: Coronary artery disease
CAC: Coronary artery calcium
SPECT: Single-photon emission computed tomography
PET/CT: Positron emission tomography/computed tomography
MPI: Myocardial perfusion imaging
MPR: Myocardial perfusion reserve
NCCT: Noncontrast computed tomography
