# Supplementary material for: Combined evaluation of CAC score and myocardial perfusion imaging in patients at risk of cardiovascular disease: where are we and what do the data say
Source: J Nucl Cardiol. 2023 May 10;30(6):2349–60. doi: 10.1007/s12350-023-03288-2 (PMC10682302; doi:10.1007/s12350-023-03288-2)
Supplement: Supplementary file 1 — Supplementary file1 (PPTX 209 KB) [file 12350_2023_3288_MOESM1_ESM.pptx]

## Slide 1
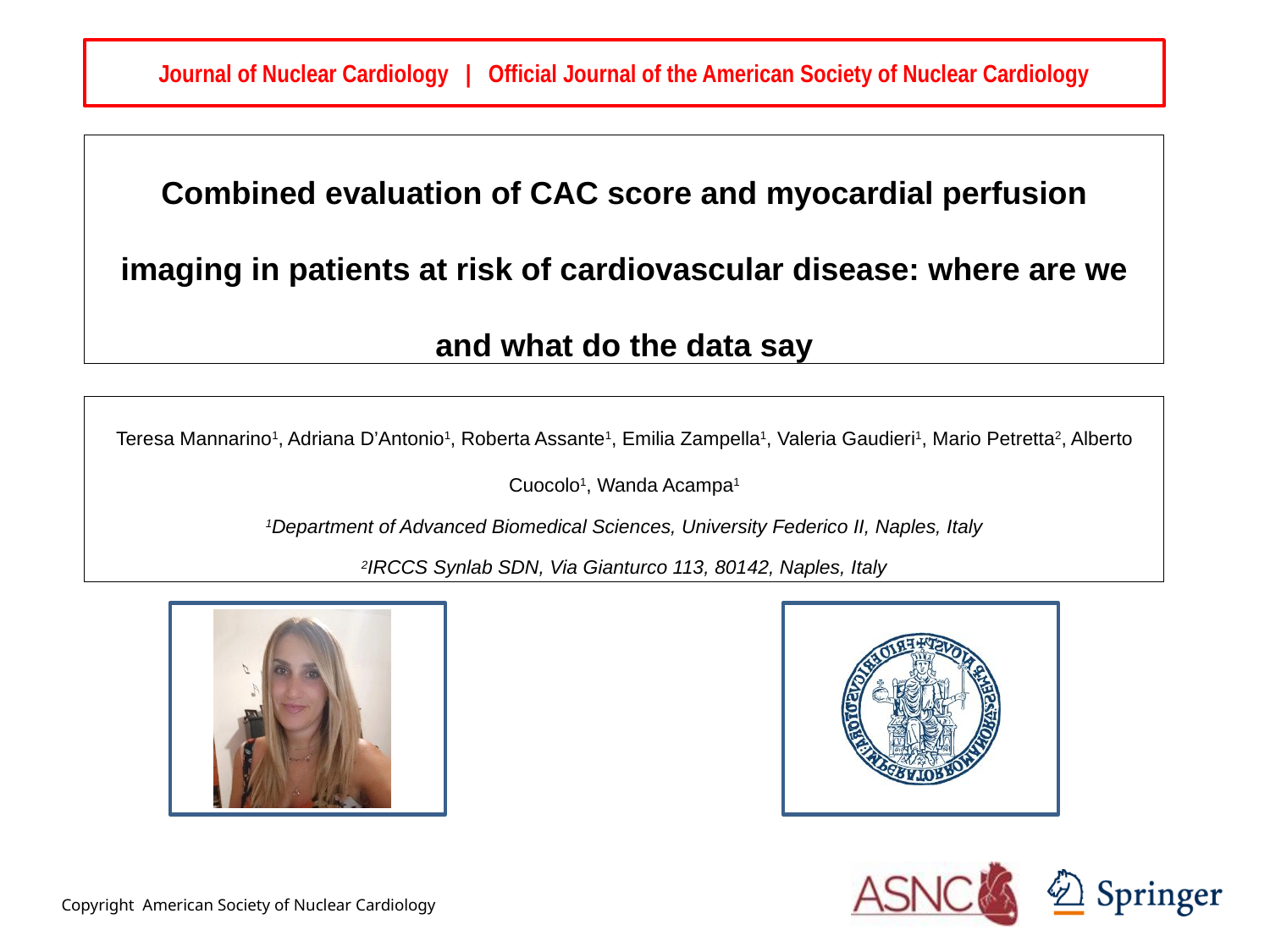

Journal of Nuclear Cardiology | Official Journal of the American Society of Nuclear Cardiology
# Combined evaluation of CAC score and myocardial perfusion imaging in patients at risk of cardiovascular disease: where are we and what do the data say
Teresa Mannarino1, Adriana D’Antonio1, Roberta Assante1, Emilia Zampella1, Valeria Gaudieri1, Mario Petretta2, Alberto Cuocolo1, Wanda Acampa1
1Department of Advanced Biomedical Sciences, University Federico II, Naples, Italy
2IRCCS Synlab SDN, Via Gianturco 113, 80142, Naples, Italy
Copyright American Society of Nuclear Cardiology

## Slide 2
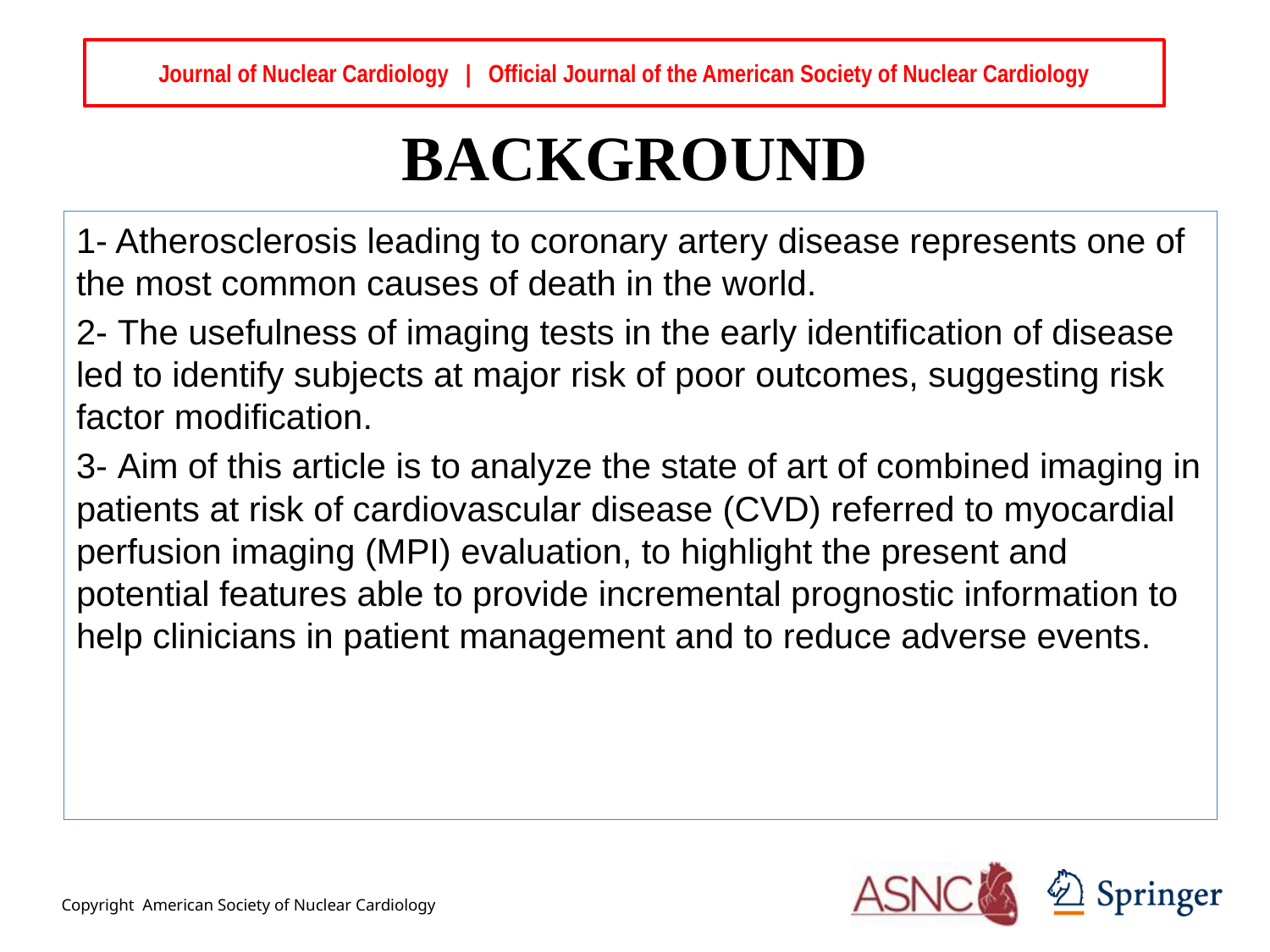

Journal of Nuclear Cardiology | Official Journal of the American Society of Nuclear Cardiology
# BACKGROUND
1- Atherosclerosis leading to coronary artery disease represents one of the most common causes of death in the world.
2- The usefulness of imaging tests in the early identification of disease led to identify subjects at major risk of poor outcomes, suggesting risk factor modification.
3- Aim of this article is to analyze the state of art of combined imaging in patients at risk of cardiovascular disease (CVD) referred to myocardial perfusion imaging (MPI) evaluation, to highlight the present and potential features able to provide incremental prognostic information to help clinicians in patient management and to reduce adverse events.
Copyright American Society of Nuclear Cardiology

## Slide 3
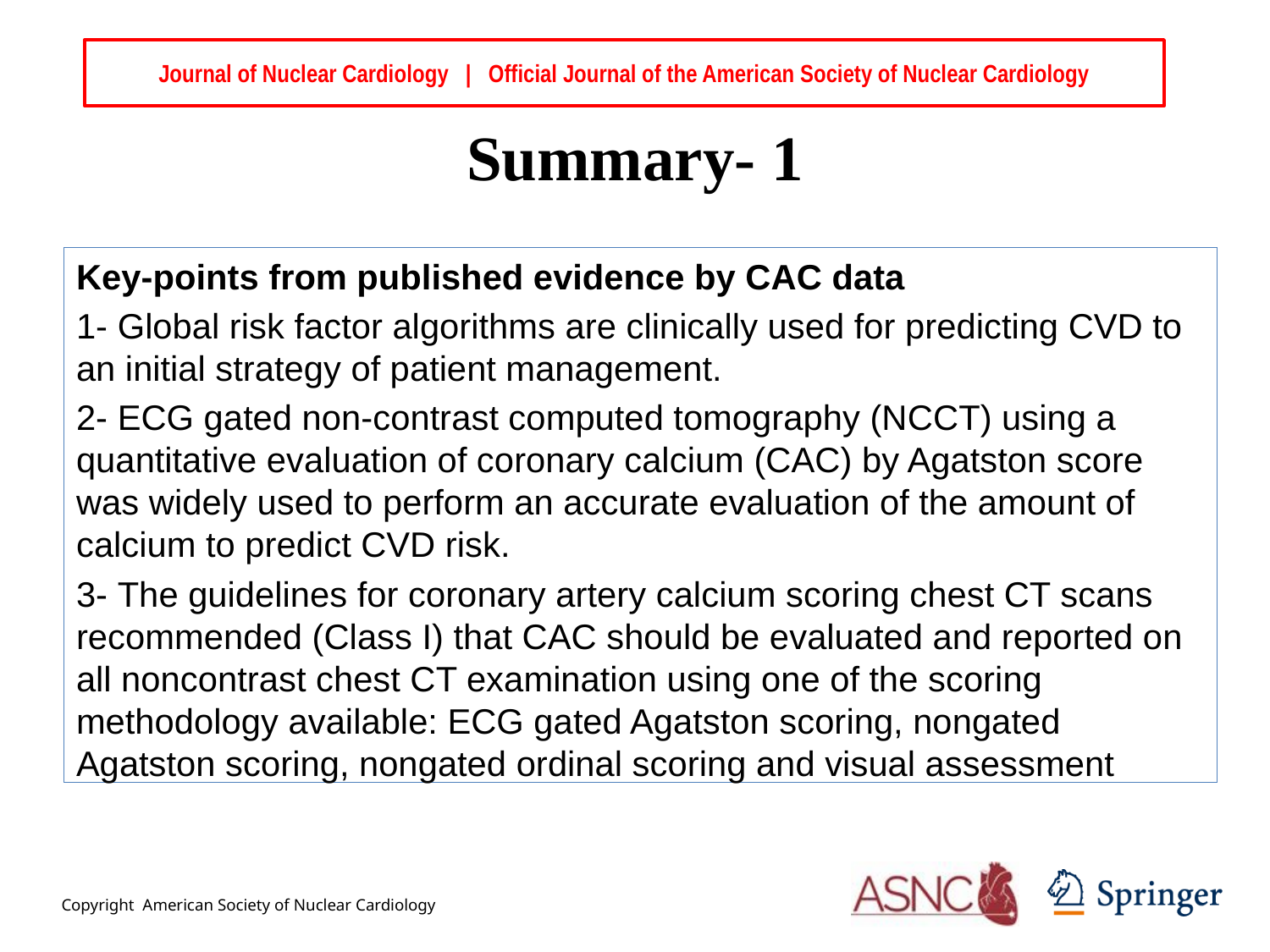

Journal of Nuclear Cardiology | Official Journal of the American Society of Nuclear Cardiology
# Summary- 1
Key-points from published evidence by CAC data
1- Global risk factor algorithms are clinically used for predicting CVD to an initial strategy of patient management.
2- ECG gated non-contrast computed tomography (NCCT) using a quantitative evaluation of coronary calcium (CAC) by Agatston score was widely used to perform an accurate evaluation of the amount of calcium to predict CVD risk.
3- The guidelines for coronary artery calcium scoring chest CT scans recommended (Class I) that CAC should be evaluated and reported on all noncontrast chest CT examination using one of the scoring methodology available: ECG gated Agatston scoring, nongated Agatston scoring, nongated ordinal scoring and visual assessment
Copyright American Society of Nuclear Cardiology

## Slide 4
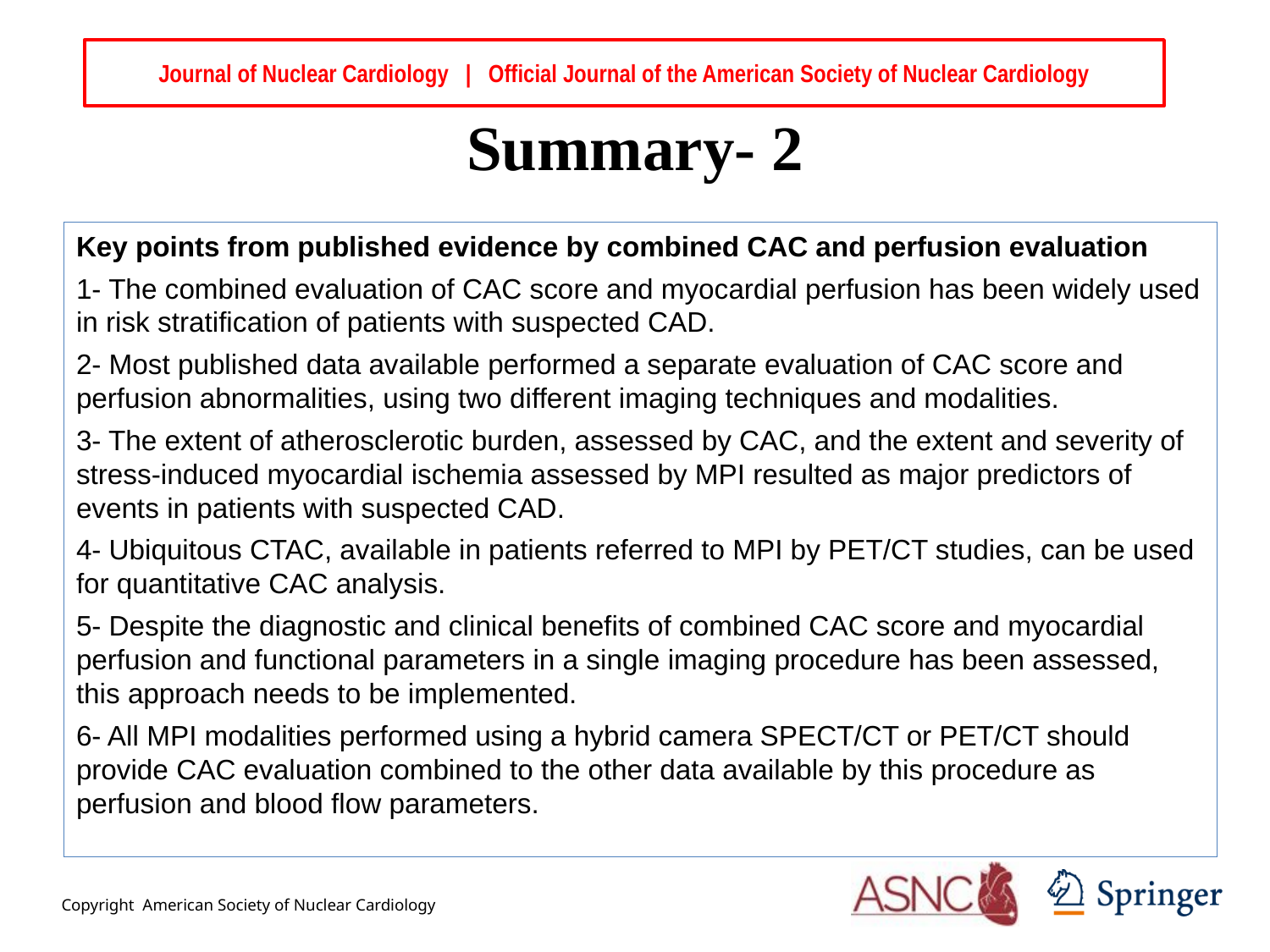

Journal of Nuclear Cardiology | Official Journal of the American Society of Nuclear Cardiology
# Summary- 2
Key points from published evidence by combined CAC and perfusion evaluation
1- The combined evaluation of CAC score and myocardial perfusion has been widely used in risk stratification of patients with suspected CAD.
2- Most published data available performed a separate evaluation of CAC score and perfusion abnormalities, using two different imaging techniques and modalities.
3- The extent of atherosclerotic burden, assessed by CAC, and the extent and severity of stress-induced myocardial ischemia assessed by MPI resulted as major predictors of events in patients with suspected CAD.
4- Ubiquitous CTAC, available in patients referred to MPI by PET/CT studies, can be used for quantitative CAC analysis.
5- Despite the diagnostic and clinical benefits of combined CAC score and myocardial perfusion and functional parameters in a single imaging procedure has been assessed, this approach needs to be implemented.
6- All MPI modalities performed using a hybrid camera SPECT/CT or PET/CT should provide CAC evaluation combined to the other data available by this procedure as perfusion and blood flow parameters.
Copyright American Society of Nuclear Cardiology

## Slide 5
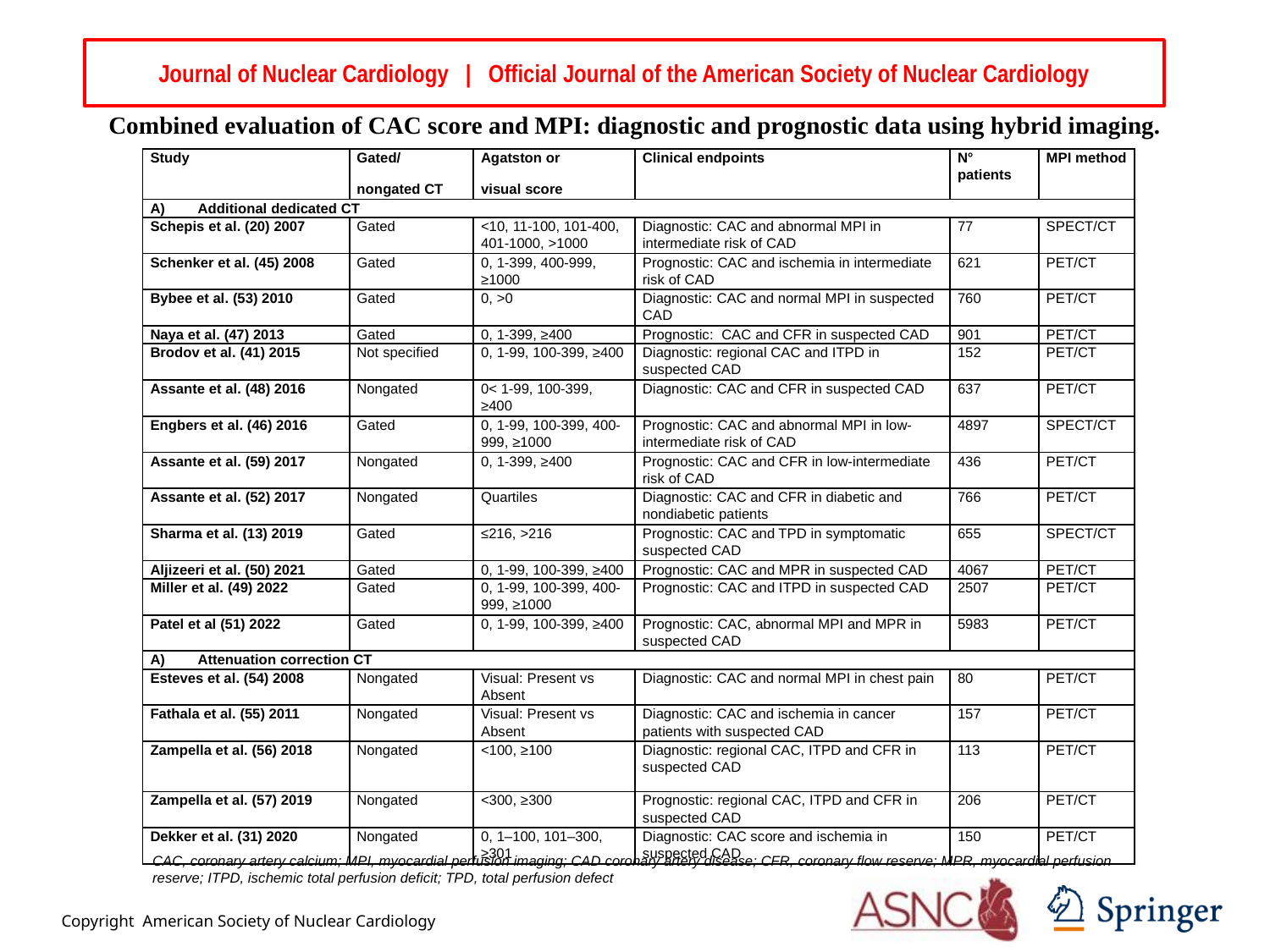

Journal of Nuclear Cardiology | Official Journal of the American Society of Nuclear Cardiology
# Combined evaluation of CAC score and MPI: diagnostic and prognostic data using hybrid imaging.
| Study | Gated/ nongated CT | Agatston or visual score | Clinical endpoints | N° patients | MPI method |
| --- | --- | --- | --- | --- | --- |
| Additional dedicated CT | | | | | |
| Schepis et al. (20) 2007 | Gated | <10, 11-100, 101-400, 401-1000, >1000 | Diagnostic: CAC and abnormal MPI in intermediate risk of CAD | 77 | SPECT/CT |
| Schenker et al. (45) 2008 | Gated | 0, 1-399, 400-999, ≥1000 | Prognostic: CAC and ischemia in intermediate risk of CAD | 621 | PET/CT |
| Bybee et al. (53) 2010 | Gated | 0, >0 | Diagnostic: CAC and normal MPI in suspected CAD | 760 | PET/CT |
| Naya et al. (47) 2013 | Gated | 0, 1-399, ≥400 | Prognostic: CAC and CFR in suspected CAD | 901 | PET/CT |
| Brodov et al. (41) 2015 | Not specified | 0, 1-99, 100-399, ≥400 | Diagnostic: regional CAC and ITPD in suspected CAD | 152 | PET/CT |
| Assante et al. (48) 2016 | Nongated | 0< 1-99, 100-399, ≥400 | Diagnostic: CAC and CFR in suspected CAD | 637 | PET/CT |
| Engbers et al. (46) 2016 | Gated | 0, 1-99, 100-399, 400-999, ≥1000 | Prognostic: CAC and abnormal MPI in low-intermediate risk of CAD | 4897 | SPECT/CT |
| Assante et al. (59) 2017 | Nongated | 0, 1-399, ≥400 | Prognostic: CAC and CFR in low-intermediate risk of CAD | 436 | PET/CT |
| Assante et al. (52) 2017 | Nongated | Quartiles | Diagnostic: CAC and CFR in diabetic and nondiabetic patients | 766 | PET/CT |
| Sharma et al. (13) 2019 | Gated | ≤216, >216 | Prognostic: CAC and TPD in symptomatic suspected CAD | 655 | SPECT/CT |
| Aljizeeri et al. (50) 2021 | Gated | 0, 1-99, 100-399, ≥400 | Prognostic: CAC and MPR in suspected CAD | 4067 | PET/CT |
| Miller et al. (49) 2022 | Gated | 0, 1-99, 100-399, 400-999, ≥1000 | Prognostic: CAC and ITPD in suspected CAD | 2507 | PET/CT |
| Patel et al (51) 2022 | Gated | 0, 1-99, 100-399, ≥400 | Prognostic: CAC, abnormal MPI and MPR in suspected CAD | 5983 | PET/CT |
| Attenuation correction CT | | | | | |
| Esteves et al. (54) 2008 | Nongated | Visual: Present vs Absent | Diagnostic: CAC and normal MPI in chest pain | 80 | PET/CT |
| Fathala et al. (55) 2011 | Nongated | Visual: Present vs Absent | Diagnostic: CAC and ischemia in cancer patients with suspected CAD | 157 | PET/CT |
| Zampella et al. (56) 2018 | Nongated | <100, ≥100 | Diagnostic: regional CAC, ITPD and CFR in suspected CAD | 113 | PET/CT |
| Zampella et al. (57) 2019 | Nongated | <300, ≥300 | Prognostic: regional CAC, ITPD and CFR in suspected CAD | 206 | PET/CT |
| Dekker et al. (31) 2020 | Nongated | 0, 1–100, 101–300, ≥301 | Diagnostic: CAC score and ischemia in suspected CAD | 150 | PET/CT |
CAC, coronary artery calcium; MPI, myocardial perfusion imaging; CAD coronary artery disease; CFR, coronary flow reserve; MPR, myocardial perfusion reserve; ITPD, ischemic total perfusion deficit; TPD, total perfusion defect
Copyright American Society of Nuclear Cardiology

## Slide 6
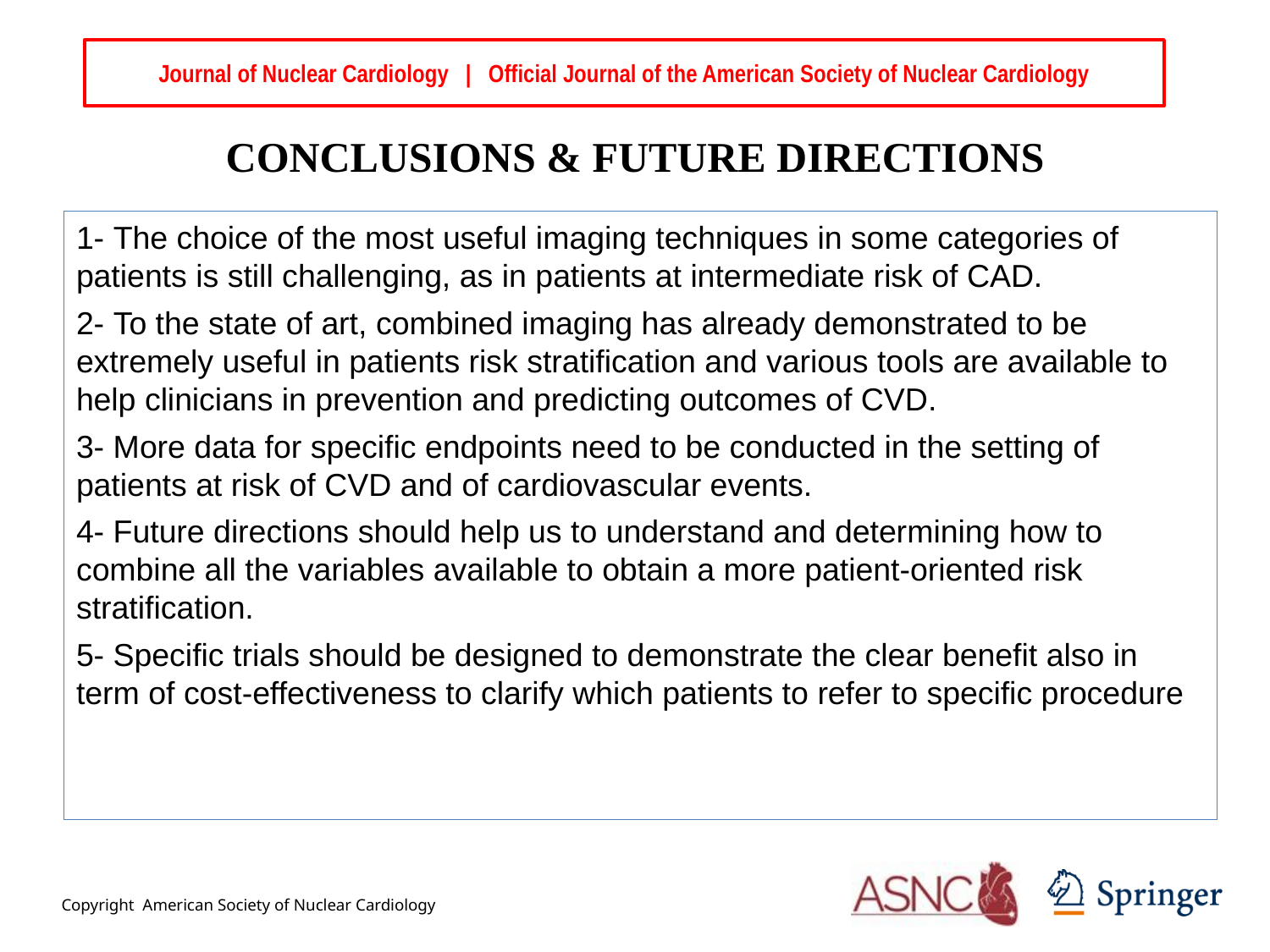

Journal of Nuclear Cardiology | Official Journal of the American Society of Nuclear Cardiology
# CONCLUSIONS & FUTURE DIRECTIONS
1- The choice of the most useful imaging techniques in some categories of patients is still challenging, as in patients at intermediate risk of CAD.
2- To the state of art, combined imaging has already demonstrated to be extremely useful in patients risk stratification and various tools are available to help clinicians in prevention and predicting outcomes of CVD.
3- More data for specific endpoints need to be conducted in the setting of patients at risk of CVD and of cardiovascular events.
4- Future directions should help us to understand and determining how to combine all the variables available to obtain a more patient-oriented risk stratification.
5- Specific trials should be designed to demonstrate the clear benefit also in term of cost-effectiveness to clarify which patients to refer to specific procedure
Copyright American Society of Nuclear Cardiology
